# Detection of feline immunodeficiency virus by neutral red-based loop-mediated isothermal amplification assay

**DOI:** 10.14202/vetworld.2024.72-81

**Published:** 2024-01-08

**Authors:** Wichayet Saejung, Kotchaporn Khumtong, Witsanu Rapichai, Siriluk Ratanabunyong, Amonpun Rattanasrisomporn, Kiattawee Choowongkomon, Oumaporn Rungsuriyawiboon, Jatuporn Rattanasrisomporn

**Affiliations:** 1 Graduate Program in Animal Health and Biomedical Sciences, Faculty of Veterinary Medicine, Kasetsart University, Bangkok, Thailand; 2 Department of Companion Animal Clinical Sciences, Faculty of Veterinary Medicine, Kasetsart University, Bangkok, Thailand; 3 Department of Biochemistry, Faculty of Science, Kasetsart University, Bangkok, Thailand; 4 Interdisciplinary of Genetic Engineering and Bioinformatics, Graduate School, Kasetsart University, Bangkok, Thailand; 5 Department of Veterinary Technology, Faculty of Veterinary Technology, Kasetsart University, Bangkok, Thailand

**Keywords:** feline immunodeficiency virus, loop-mediated isothermal amplification, molecular diagnosis, neutral red

## Abstract

**Background and Aim:**

Feline immunodeficiency virus (FIV) is a retroviral pathogen globally responsible for immunodeficiency disease in cats. However, the current diagnosis based on antibody detection has limitations and can also produce false-positive results. This study aimed to develop a one-pot loop-mediated isothermal amplification (LAMP) process integrated with neutral red (NR-LAMP) assay for detection of FIV proviral DNA.

**Materials and Methods:**

We developed a one-pot, *gag* gene-based NR-LAMP for convenient, rapid, specific, and sensitive colorimetric inspection of FIV proviral DNA.

**Results:**

The developed NR-LAMP was capable of amplifying at an optimum temperature of 65°C for 40 min. No cross-amplification was detected between FIV and other feline viruses tested, indicating the high specificity (98.44%) of the novel FIV-LAMP primer. Our NR-LAMP assay has a detection limit of 4.2 × 10^1^ copies/μL. A total of 80 clinical samples with a background of FIV infection were collected and tested using the proposed method. The NR-LAMP assay showed a high sensitivity of 100% compared to conventional polymerase chain reaction assay.

**Conclusion:**

These results support the suitability of NR-LAMP as a potential future alternative clinical molecular approach for further use in the diagnosis of FIV-infected cats.

## Introduction

In 1986, feline immunodeficiency virus (FIV) was first isolated in Northern California, USA [1]. This pathogen spreads worldwide and is specific to cat populations and has been classified as a member of the family *Retroviridae*, subfamily *Orthoretrovirinae* in the genus Lentivirus. A single-stranded RNA length of approximately 9.5 kb with a 5′-*gag*-*pol*-*env*-3′ genome orientation [2] was identified. Feline-acquired immunodeficiency syndrome is a symptomatic disease; however, it is not identical to human immunodeficiency virus (HIV), including the fact that FIV uses a different receptor to enter the cell and that cats may live for many years without treatment and without any clinical signs. In addition, it can increase the risk of opportunistic infections [3–6], neurological disorders, and tumors [7, 8]. The main transmission of viral infections occurs by biting during fighting between cats and transmission through the placenta to the offspring. Similar to HIV infection in humans, viral RNA is reverse-transcribed into double-stranded DNA through the reverse transcription process in the cytoplasm and then transported to the nucleus before integration into the host genome as proviral DNA by the viral integrase enzyme [9].

FIV infections are screened using indirect fluorescent antibody (IFA) technique or enzyme-linked immunosorbent assay (ELISA) [6, 10–16] to detect FIV antibodies. However, low antibody responses during the first 2–4 weeks of infection, including terminal disease, can lead to misinterpretation [7]. Furthermore, maternal anti-FIV antibodies in kittens and vaccinated cats can produce false-positive results [17–20], making the serological results difficult to interpret. Recently, several molecular approaches related to FIV proviral DNA inspection, especially in the subclinical phase, have been developed using conventional, nested, and quantitative polymerase chain reaction (qPCR) [13–15, 21–24]. Although it can be confirmed in certain circumstances, there are certain disadvantages, such as the need for professional technicians, expensive equipment, a long operation period, and complicated working procedures. Loop-mediated isothermal amplification (LAMP) has recently been shown to be a convenient, sensitive, and rapid method for detection at a single temperature [25–28]. In addition, agarose gel electrophoresis (AGE) and lateral flow dipstick are not required for data interpretation. During amplification, pyrophosphate tetravalent anions are generated as by-products that can react with magnesium, resulting in white precipitation [29, 30] and hydrogen ions, which predominantly affect the pH value, changing from alkaline to acidic. In our recent work, we developed a reliable colorimetric RT-LAMP assay using pH-sensitive neutral red (NR), which has a detection limit of 10^6^ lower than that of conventional PCR for feline coronavirus (FCoV) diagnosis [25].

This study aimed to develop a one-pot NR-LAMP assay for detection of FIV proviral DNA. After optimizing the NR-based isothermal amplification approach using a recombinant plasmid harboring the FIV-*gag* gene as a template, specificity was tested with other related feline viruses and the detection limit was quantified. In addition, its potential for clinical applications has also been determined.

## Materials and Methods

### Ethical approval

This study was approved by the Institutional Animal Care and Use Committee of Kasetsart University (ACKU65-VET-081), Bangkok.

### Study period and location

This study was conducted from August 2021 to March 2023 at the Kasetsart University Veterinary Teaching Hospital.

### Construction of standard plasmid WS-gag

Total RNA was isolated from FIV vaccine (Fel-O-Vax FIV^®^, Fort Dodge Animal Health, USA) using E.Z.N.A. Viral RNA Kit (Omega Bio-Tek, Norcross, GA, USA) according to the manufacturer’s instructions. cDNA synthesis reaction was carried out in a total volume of 20 μL by mixing Oligo(dT)_18_ primer, RNase-free water, 1× reaction buffer, 1 U RiboLock RNase Inhibitor (Thermo Scientific, Wilmington, NC, USA), 1 mM dNTP mix, 0.1 U RevertAid-M-MuLV RT (Thermo Scientific), and 10 ng/μL FIV RNA template. The reactions were incubated at 42°C for 60 min and 70°C for 5 min. FIV cDNA was further used as a template for PCR amplification.

The FIV-*gag* gene was amplified and cloned into the pGEM-T vector to construct the standard plasmid. Briefly, PCR amplification of an approximately 200-bp DNA fragment of the FIV-*gag* gene was carried out using the F3 and B3 outer primers (Table-1). The PCR reaction was performed in a 25 μL reaction mixture consisting of 1× DreamTaq Green PCR Master Mix (Thermo Scientific), 0.24 μM each of outer primers, and 1 μL of templated FIV cDNA. The reaction was initially denatured at 94°C for 3 min, followed by 40 cycles of 94°C for 30 s, 52°C for 30 s, 72°C for 40 s, and a final extension at 72°C for 5 min. An approximately 200-bp amplified fragment containing the *gag* gene was then cloned into the pGEM-T vector, yielding the recombinant plasmid WS-*gag*, which was transformed into competent *Escherichia coli* DH5α according to the manufacturer’s instructions (Promega, Madison, WI, USA). A positive clone was selected on Luria Bertani (LB) agar (Himedia, Mumbai, India) containing 0.1 mg/mL ampicillin after overnight culture, confirmed by colony PCR, and used for plasmid extraction, which was applied as the standard DNA template in subsequent experiments.

**Table-1:** Primers used for detection of FIV-*gag* gene using LAMP and polymerase chain reaction (patent application no. 2303001892).

Primer name	Position	Sequence (5’ → 3’)
F3	1230–1252	AATATTGGATGAAAGCTTAAAGC
B3	1411–1430	GCCAATTTTCCTAATGCCTC
FIP (F1c–F2)	1253–1274	ACCCATAATTTCTGCTGCAGTAAAA-AACTGACAGCAGAATATGATCG
BIP (B1c–B2)	1391–1410	AGGATTAACTCAAGAACAACAAGCA-GAGATACCATGCTCTACACT
Loop F	1276–1294	GAGCATCAGGGGGATGTGT
Loop B	1338–1364	GCAAGATTTGCACCAGCTAGG

LAMP=Loop-mediated isothermal amplification

### LAMP primer design

**Figure-1:**
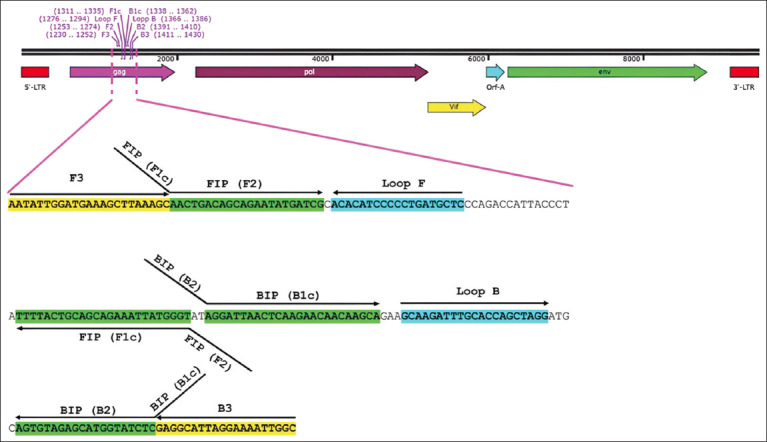
FIV genomic orientation and location of LAMP primer sets targeting *gag*-FIV sequence. Positions for two outer (F3 and B3), two inner (FIP and BIP), and two loop (LF and Luria Bertani) primers are indicated in yellow color, green color, and sky-blue color, respectively. FIP is a hybrid primer consisting of the F1c sequence and the F2 sequence, and BIP is a hybrid primer consisting of the B1c sequence and the B2 sequence. Arrows indicate the extension direction. FIV=Feline immunodeficiency virus, LAMP=Loop-mediated isothermal amplification.

Based on the conserved region of FIV-*gag* gene, several sequences of FIV obtained from GenBank database with accession numbers of AY684181, AF531070, AF531068, D37820, DQ365591, DQ365590, EU025246, GQ422127, GQ339854, GQ339852, GQ339868, GQ339820, GQ339834, GQ339835, GQ339839, GQ339819, L06136, GQ339862, GQ339843, JF411740, KM880120, KM880117, M25381, MF352016, NC_001482, and X57002 were performed multiple sequence alignment using MEGA X software (https://www.megasoftware.net/). An accession number of NC_001482 was used as a representative sequence for LAMP primer design using Primer Explorer version 5 (http://primerexplorer.jp/lampv5e/index.html) (Figure-1). In this study, we utilized a set of primers consisting of two outer primers (F3 and B3), two inner primers (forward inner primer [FIP] and backward inner primer [BIP]), and two loop primers (loop forward [LF] and loop backward [LB]). The primer sequences are listed in Table-1.

### LAMP reaction and optimization

The colorimetric LAMP reaction was carried out in a total volume of 25 μL containing 10× LAMP buffer (100 mM KCl, 100 mM (NH_4_)_2_SO_4_, and 10% Tween 20 pH 8.8), 25 mM MgCl_2_, 10 mM dNTPs (Thermo Scientific), 10× LAMP primer mix (0.2 μM each of F3 and B3, 1.6 μM each of FIP and BIP, and 0.4 μM each of Loop F and Loop B), 2.5 mM NR (Invitrogen; Waltham, MA, USA), 8 U/μL *Bst* 2.0 Warmstart DNA polymerase (New England BioLabs; Ipswich, MA, USA), and 1 ng/μL DNA template. RNase-free water was used as a negative control in all experiments. The LAMP amplification was incubated at 65°C for 40 min and inactivated at 85°C for 5 min using a T100 thermal cycler (Bio-Rad, Hercules; CA, USA), after which it was visualized using the naked eye. An amplification reaction color change from yellow to pink indicated a positive reaction, while the color remaining yellow indicated a negative reaction. The optimum conditions for the NR-LAMP reaction were determined based on optimizing from ranges in temperature (60–70°C), incubation time (10–60 min), MgCl_2_ concentration (2–10 mM), and dNTP concentration (0.6–2.2 mM).

### Specificity of LAMP assay

The LAMP specificity assay was performed under optimum conditions using viral DNA/RNA obtained from clinical samples with feline calicivirus (FCV), FCoV, feline leukemia virus (FeLV), feline herpesvirus (FHV), FIV, and feline panleukopenia virus (FPV) infections. FCoV DNA with positive ORF1ab PCR results was kindly provided by Rapichai *et al*. [25]. The feline clinic of Kasetsart University Veterinary Teaching Hospital provided the stock of other pathogenic genetic materials including FCV, FeLV, FHV, FIV, and FPV. In addition, uninfected Crandell-Rees Feline Kidney (CRFK) cells and whole blood were used as internal controls. The standard WS-*gag* plasmid was used as the positive control, and RNase-free water was used as the negative control. A CFX96 Touch Real-Time PCR Detection System (Bio-Rad) was used to detect fluorometric amplification signals, and the LAMP products were visualized based on electrophoresis and naked eye visualization.

### Sensitivity of qPCR and LAMP assay

The analytical sensitivity of our developed LAMP assays was determined from the lowest concentration of 10-fold serially diluted standard plasmid WS-*gag* compared with that of qPCR. Briefly, 10-fold serial dilutions of recombinant plasmid WS-*gag* were prepared to obtain varying concentrations in the range 10^6^–10^0^ copies/μL. For qPCR, 25 μL reaction mixture was carried out containing 1× Maxima SYBR Green qPCR Master Mix (Thermo Scientific), 0.6 μM each of the F3 and B3 primers, and 1 μL of each diluted standard plasmid WS-*gag*. The qPCR conditions were initial denaturation at 94°C for 3 min, followed by 40 cycles at 94°C for 30 s, 52°C for 30 s, 72°C for 40 s, and a final extension at 72°C for 5 min. The amplification product was monitored at each elongation step using a CFX96 touch real-time PCR detection system instrument (Bio-Rad). For the LAMP evaluation, both the fluorescent signal and colorimetric detection were monitored using the same device at 65°C for 60 min through the Texas red channel, which allowed us to determine the limit of detection (LOD) of our developed NR-based LAMP FIV. All samples were amplified in triplicate. The recombinant plasmid concentration was calculated and expressed as the viral copy number per microliter using the following equation [31]:


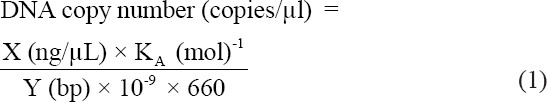


Where X is the recombinant plasmid concentration, Y is the number of base pairs in the vector, and K_A_ is Avogadro’s constant (6.022 × 10^23^).

### Sample extraction and clinical validation of the NR-LAMP assay

In total, 80 whole blood samples were collected from cats with suspected FIV infection (showing signs of weight loss, lethargy, fever, gingivitis, and stomatitis) in Kasetsart University Veterinary Hospital, Thailand. Proviral DNA was extracted using a GF-1 viral nucleic acid extraction kit (Vivantis; Subang Jaya, Selangor, Malaysia) according to the manufacturer’s instructions and then quantified using a NanoDrop spectrophotometer (Thermo Scientific). Extracted FIV proviral DNA was used as a template for LAMP and PCR analysis. Recombinant plasmid WS-*gag* was used as the positive control and RNase-free water was used as the negative control. The reaction mixtures of conventional PCR contained 1× DreamTaq green PCR master mix (2×), 0.24 μM each F3 and B3 primers, 1 μL proviral DNA template, and nuclease-free water in a final volume of 25 μL. The PCR conditions were initial denaturation at 94°C for 3 min; 40 cycles of 94°C for 30 s, 52°C for 30 s, 72°C for 40 s, and a final extension at 72°C for 5 min. Conventional PCR was performed using a T100 thermal cycler (Bio-Rad). The PCR products were analyzed based on electrophoresis on a 1.5% (w/v) agarose gel and visualized using a UV transilluminator. The colorimetric LAMP reaction was carried out in a total volume of 25 μL containing 10× LAMP buffer (100 mM KCl, 100 mM (NH_4_)_2_SO_4_, and 10% Tween 20 pH 8.8), 25 mM MgCl_2_, 10 mM dNTPs (Thermo Scientific), 10× LAMP primer mix (0.2 μM each of F3 and B3, 1.6 μM each of FIP and BIP, and 0.4 μM each of Loop F and Loop B), 2.5 mM NR (Invitrogen), 8 U/μL *Bst* 2.0 Warmstart DNA polymerase (New England BioLabs), and 1 ng/μL DNA template. RNase-free water was used as a negative control in all experiments. The LAMP amplification was incubated at 65°C for 40 min and inactivated at 85°C for 5 min using a T100 thermal cycler (Bio-Rad), after which, it was visualized using the naked eye.

### Statistical analysis

The diagnostic sensitivity and specificity of the clinical samples were determined using LAMP assay and compared with those of PCR assay. The FIV-*gag* gene was identified using both LAMP assay and conventional PCR, and the results were subsequently calculated into a two-by-two table. To determine the effectiveness of LAMP for FIV detection, sensitivity, and specificity were considered as percentages, and Cohen’s kappa coefficient (ĸ) value was used to analyze the agreement between assays.

## Results

### Optimization of colorimetric LAMP reaction with NR as an indicator dye

**Figure-2:**
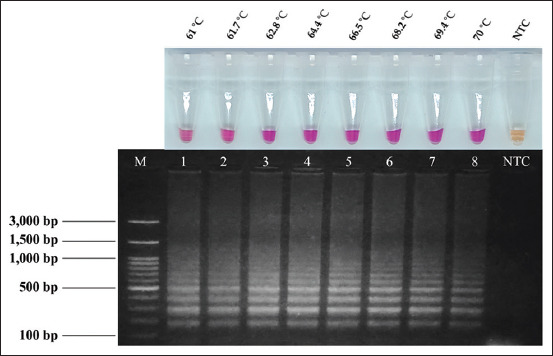
Temperature optimization of neutral red-loop-mediated isothermal amplification assay was detected by naked eyes and by agarose gel electrophoresis. Pink color shows positive reaction and yellow color shows negative reaction. Lane M, 100 bp Ladder DNA Marker III (Yeastern Biotech, New Taipei City, Taiwan), lane 1–8, temperature amplification at 61–70°C, and Lane NTC, negative control.

Using NR dye as an indicator in the positive colorimetric LAMP reaction for visualizing the color change from yellow to pink, the effect of temperature on the NR-LAMP assay was optimized in the range 61–70°C. As shown in Figure-2, the NR-LAMP assay yielded positive results with a pink color over a wide temperature range (61–70°C) that was apparent to the naked eye. Because 65°C is the optimum temperature for an isothermal enzyme, it was chosen throughout the following experiments.

**Figure-3:**
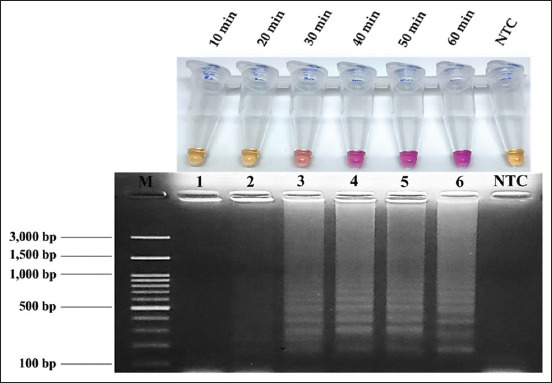
Amplification time optimization of neutral red-loop-mediated isothermal amplification assays that were detected by naked eyes and by agarose gel electrophoresis. Pink color shows positive reaction and yellow color shows negative reaction. Lane M, 100 bp Ladder DNA Marker III (Yeastern Biotech, New Taipei City, Taiwan), lane 1–6, amplification times at 65°C for 10–60 min, and lane no template control (NTC), negative control.

To identify the most suitable amplification time under optimum temperature, the incubation period of the NR-LAMP reactions was investigated. As shown in Figure-3, the pink color appeared as quickly as 30 min after incubation. However, an intense pink color could be clearly observed between 40 and 60 min, indicating that 40 min was the optimal amplification time for the subsequent experiments. To determine the effect of MgCl_2_ concentration on the NR-based reaction, it was apparent that 6 mM MgCl_2_ produced an intense pink color in the tube, whereas 4 and 8 mM MgCl_2_ produced a faint pink color in the tube (Figure-S1). With regard to the effect of dNTPs, 1.4 mM alone produced a pink color (Figure-S2).

### Analytical specificity of colorimetric LAMP assay for FIV detection

**Figure-4:**
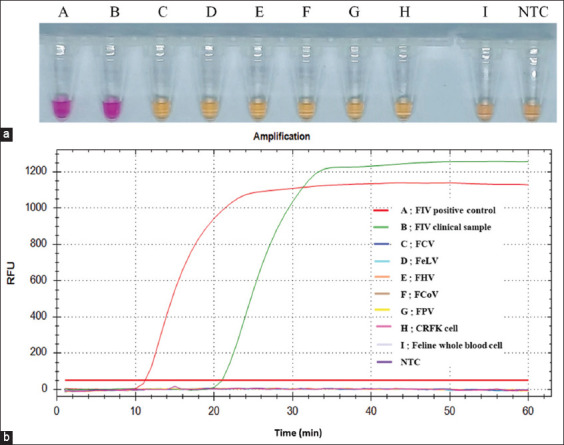
Specificity of LAMP primers for FIV detection. (a) Specificity analysis of LAMP assay using neutral red indicator dye. (b) Specificity analysis of LAMP assays using fluorescent signal. Tube A: positive control (standard plasmid WS-*gag*), Tube B: FIV clinical sample, Tube C: FCV, Tube D: FeLV, Tube E: FHV, Tube F: FCoV, Tube G: FPV, Tube H: CRFK cells, Tube I: feline whole blood and NTC: No template control. The amplification plot on the Y-axis represents the relative fluorescence unit (RFU) value, where the X-axis represents the amplification time (min). FIV=Feline immunodeficiency virus, LAMP=Loop-mediated isothermal amplification, FCV=Feline calicivirus, FeLV=Feline leukemia virus, FHV=Feline herpesvirus, FCoV=Feline coronavirus, FPV=Feline panleukopenia virus.

LAMP primer specificity is crucial for accuracy in amplifying genetic material from feline viruses, including FCV, FCoV, FeLV, FHV, FIV, and FPV. We designed primers based on the conserved FIV-*gag* gene and tested them against other feline viruses, using uninfected CRFK cells and feline whole blood as internal controls of feline housekeeping genes [32, 33]. Before specificity analysis, it was cross-validated with reference sequences of other feline viruses, including FCV (KP987265), FCoV (DQ848678), FeLV (MT129531), FHV (NC_013590), FIV (M25381), and FPV (MG924893). As expected, *in silico* primer analysis revealed no cross-amplification with other feline viruses (data not shown). As shown in Figure-4a, only the FIV clinical sample exhibited a pink color similar to that of the positive control, whereas the amplification results showed that none of the feline virus reactions and feline housekeeping genes were amplified under the optimum conditions.

Furthermore, the NR-LAMP fluorometric signal was utilized to affirm FIV specificity (Figure-4b), showing that only the FIV clinical sample and positive control increased the amplification curve, with no signal from other feline viruses and feline housekeeping genes. In addition, AGE revealed only the DNA ladder pattern of pinkish reaction from FIV clinical sample tube and positive control tube (Figure-S3). The lowest DNA band was excised from the agarose gel, purified, and nucleotide sequencing was performed to confirm whether the tested clinical sample was obtained from an FIV-infected cat. After similarity analysis, our results showed 99% identity with accession no. KM880120 of FIV isolate BEHEramo (Figure-S4). In summary, the proposed FIV NR-LAMP assay showed no cross-amplification with the five feline viruses and feline housekeeping genes, suggesting that our designed primers had high specificity and the potential to be applicable at the clinical level.

### Sensitivity comparison of NR-LAMP and qPCR assay for FIV detection

**Figure-5:**
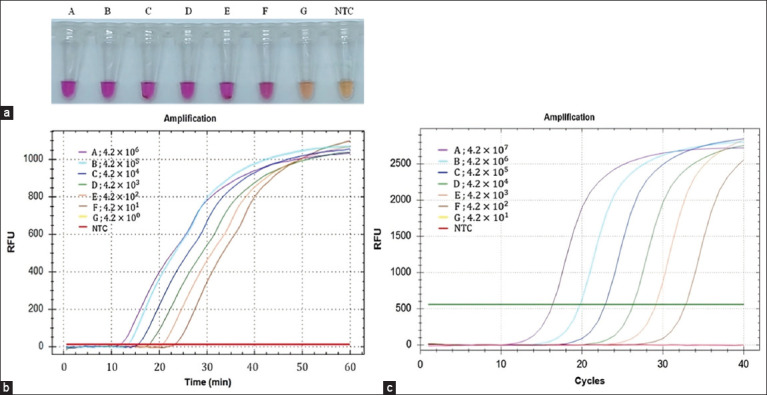
Comparative sensitivity of LAMP and qPCR for feline immunodeficiency virus detection. (a) Sensitivity of colorimetric NR-LAMP assay. (b) Sensitivity of colorimetric NR-cLAMP assay using fluorescent signal. The amplification plot on the Y-axis represents the RFU value, whereas the X-axis represents the amplification time (min). (c) Sensitivity of qPCR. Tube A: 4.2× 10^6^ copies/μL; Tube B: 4.2× 10^5^ copies/μL; Tube C: 4.2× 10^4^ copies/μL; Tube D: 4.2× 10^3^ copies/μL; Tube E: 4.2 × 10^2^ copies/μL; Tube F: 4.2 × 10^1^ copies/μL; Tube G: 4.2× 10^0^ copies/μL; NTC: No template control. The amplification plot on the Y-axis represents the RFU value, where the X-axis represents the amplification cycle. qPCR=Quantitative polymerase chain reaction, NR-LAMP=Neutral red-loop-mediated isothermal amplification.

For determining the LOD for FIV proviral DNA detection, both assays (NR-LAMP and qPCR) were examined with the same concentration series of 10-fold serially diluted standard plasmid WS-*gag*, ranging from 4.2 × 10^0^ to 4.2 × 10^6^ copies/μL. The lowest copy number that could be detected by the naked eye (Figure-5a) and by AGE (Figure-S5) consistent with a fluorescent signal (Figure-5b) was 4.2 × 10^1^ copies/μL. Furthermore, the minimum concentration that could be observed using qPCR was 4.2 × 10^2^ copies/μL (Figure-5c) and by AGE (Figure-S6). Thus, the LOD of the proposed assay was 10-fold lower than that of qPCR, as well as being less time-consuming than qPCR, by approximately 1 h. These results indicated the high efficiency of NR-LAMP for FIV diagnosis.

### Clinical validation of the NR-LAMP assay for FIV detection

Practical feasibility of NR-LAMP use in clinical laboratories was investigated. In total, 80 clinical samples from FIV cats deemed to be suspicious were examined. The results of NR-LAMP were compared with the conventional PCR assay. As summarized in Table-2, using conventional PCR as the reference standard, FIV proviral DNA was detected in 16 of 17 true-positive samples by both NR-LAMP and PCR assay. One of the 17 results was false positive, whereas 63 of 80 samples were true negative. The diagnostic sensitivity of NR-LAMP for FIV detection was 100% (95% confidence interval [CI], 79.41–100.0), and the diagnostic specificity was 98.44% (95% CI, 91.60–99.96). The level of agreement between conventional PCR and NR-LAMP assay was 0.96, which indicated perfect agreement between the two assays. In addition, the 1 false positive was subjected to DNA sequencing. After similarity analysis, these samples were identical to the FIV-*gag* gene of accession number M25381. This result confirms the potential of the developed LAMP method as a reliable alternative for FIV detection. Furthermore, these results validated the effectiveness of the LAMP assay in accurately identifying FIV in whole blood samples.

**Table-2:** Comparative analysis of conventional PCR and LAMP for detecting FIV proviral DNA.

NR-LAMP	Conventional PCR			Sensitivity (%) (95% CI)	Specificity (%) (95% CI)	κ-value

	Positive	Negative	Total			
Positive	16	1	17	100 (79.41–100.00)	98.44 (91.60–99.96)	0.96
Negative	0	63	63			
Total	16	63	80			

CI=Confidence interval, κ=Kappa coefficient, PCR=Polymerase chain reaction, FIV=Feline immunodeficiency virus, NR-LAMP=Neutral red-loop-mediated isothermal amplification.

## Discussion

Serological assays, such as ELISA and IFA, targeting antibodies against FIV, are widely used in veterinary clinics and research settings due to their convenience in handling large sample sizes. Infected cats can be identified when antibodies against FIV develop within a few weeks after infection. However, in some cases of FIV-positive cats, serological tests may produce false-positive results. Therefore, confirmatory molecular methods are required to ascertain the presence of the virus. Various molecular detection techniques, including conventional PCR, nested PCR, and qPCR, can be utilized for the detection of FIV proviral DNA [13–15, 21, 22]. LAMP has been developed by Notomi *et al*. [29, 34–36] as a simple and rapid gene amplification approach. Hybrid FIP and BIP primers can create dumbbell-like DNA structure, generating stem-loop formation at both ends, which were then used as the starting material for the second stage of LAMP reaction. FIP/BIP hybrid primers used in this technique are essential for increasing specificity and amplification efficiency under isothermal conditions due to the employment of four primers targeting six separate regions on the DNA sequence. LAMP, a DNA amplification method [34], has gained widespread use in identifying bacterial, parasitic, and viral infections [25, 26, 30, 37–46]. In addition, the simplicity, sensitivity, and specificity of this method make it particularly promising for alternative molecular detection assays associated with other serological assays [47]. In contrast to PCR, which requires specialized thermal cycling equipment, LAMP can be performed at a single temperature without the need for expensive devices [30].

Various techniques have recently been proposed to facilitate visualization of LAMP outcomes. One such approach entails AGE, where distinctive ladder-like patterns are displayed, indicating products of varying DNA bands. Alternative methods include turbidity measurement [17, 19], implementation of fluorescent dyes, such as SYBR green [30, 48] and pico green [44, 49], as well as utilization of metal-sensitive dyes, such as calcein [36], malachite green [43, 46], and hydroxy naphthol blue [45, 50, 51].In addition, pH-sensitive dyes, such as cresol red [28], phenol red [52], xylenol orange [41], and NR [25, 26, 53, 54], have been investigated for observing color changes resulting from a decrease in pH caused by the release of protons during the amplification reaction [55]. Notably, NR dyes facilitate easy visualization of color changes from yellow to pink with the naked eye, eliminating the need for complicated procedures such as AGE. In addition, this developed approach reduces the risk of cross-contamination due to repeated opening of the tube.

The optimum parameters for colorimetric LAMP reactions for the detection of a positive colorimetric reaction vary according to the different LAMP protocols, such as temperature, amplification time, and concentration of components. The present study is the first attempt to develop a LAMP assay for detecting FIV using a LAMP primer set targeting the conserved *gag* genes of FIV [56]. The results showed that the developed technique for FIV detection using NR-LAMP was successful. The colorimetric assay exhibited the capability to diagnose FIV showing yellow-to-pink color within 40–60 min. In addition, this developed NR-LAMP assay for the detection of FIV revealed high specificity by amplifying only FIV, showed no cross-reactivity among the feline viruses studied, and was 10 times more sensitive than qPCR. These results demonstrated that our new primers effectively enhanced the sensitivity and specificity of the reaction and enabled rapid and simple detection of FIV provirus using the developed NR-LAMP assay.

We validated the applicability of clinical samples and colorimetric LAMP assay against conventional PCR using 80 whole blood samples. Overall, there was a kappa value of 0.96 between the two tests. In addition, the two assays showed perfect agreement for FIV detection. These results indicate that the developed NR-LAMP assay has the potential to be an efficient, rapid, and user-friendly tool. Compared to typical PCR assays with a higher detection limit, false-positive results may occur when NR-LAMP is used in veterinary practice. This is likely due to the minimal quantity of proviral DNA, sample quality, time of sample collection from disease onset, and the use of six novel LAMP primers specific to the FIV *gag* gene [57, 58]. To confirm FIV-infected cats, nucleotide sequencing is required. Furthermore, the developed LAMP assay offers high sensitivity, specificity, and visual detection of the LAMP products despite the requirement for proviral DNA extraction. Therefore, this molecular method should be considered for the clinical detection of FIV, offering a viable alternative for veterinarians.

FIV has been classified in the lentivirus genus and causes AIDS-like immunodeficiency disease in felines. Previously, one approach used animal models to investigate pragmatic solutions for HIV infection in humans, based on a genetically comparable virus that could produce an identical disease as close to as possible, having a similar temporal pathogenesis, and be easily available to all researchers [59, 60]. Cats infected with FIV meet all these criteria. Thus, NR-LAMP would be an alternative reliable and cost-effective model to pave the way for HIV diagnosis due to its low risk of false-positive results for FIV detection.

In addition, this procedure could be adapted to effectively identify other pathogens, making it an invaluable tool for future detection efforts. However, uncomplicated and resilient DNA extraction methods need to be integrated to increase their practicality. Notably, compared with PCR, LAMP showed reduced susceptibility to sample inhibition and allowed the omission of the DNA extraction step. Consequently, the viability of minimally processed samples to optimize the efficiency and usability of NR-LAMP assays should be prioritized for future research.

## Conclusion

The novel colorimetric LAMP assay for FIV detection developed here was sensitive, specific, rapid, convenient, and observable by the naked eye immediately after amplification, presenting a novel LAMP primer with levels of sensitivity and specificity consistent with conventional PCR assays. Therefore, this novel method could be used as an alternative diagnostic technique to prevent and control FIV at the clinical level.

### Data Availability

All supplementary data including Figures-S1 to S6 can be available from the corresponding author on a reasonable request.

### Authors’ Contributions

WS, WR, OR and JR: Designed the study. WS, KK, WR, AR, OR, SR, and JR: Collected the data and performed the analysis. KC: Software analysis. WS and WR: Drafted the manuscript. WR, AR, OR, and JR: Writing, reviewing and editing of the manuscript. All authors have read, reviewed, and approved the final manuscript.
